# The association between confidence in health system and self-rated health: from the perspective of regional comparison

**DOI:** 10.3389/fpubh.2025.1656639

**Published:** 2025-10-10

**Authors:** Zhaoxi Wang, Kairan Zhang

**Affiliations:** ^1^School of Public Administration, Northwest University, Xi’an, Shaanxi, China; ^2^School of Political Science and Public Administration, Northwest University of Political Science and Law, Xi’an, Shaanxi, China

**Keywords:** confidence in health system, affordability confidence, accessibility confidence, self-rated health, regional comparison

## Abstract

**Background:**

Universal health coverage (UHC) aims to ensure that all people can access quality health services without financial hardship, and thereby improve health outcomes. Confidence in the health system reflects the individuals’ belief that they can obtain or afford care when ill. This study examined the association between health system confidence and self-rated health (SRH) across urban–rural and regional (western, middle, and eastern) areas of China.

**Method:**

Data were drawn from two waves of the Chinese General Social Survey that included 6,481 participants. Ordered logistic regression was used to assess the association between health system confidence and SRH. Subsample analyses and interaction models were used to explore regional heterogeneity.

**Results:**

Both self-rated health (SRH) and confidence in obtaining and affording healthcare were higher in urban and eastern regions. Greater confidence in the health system was positively associated with better SRH. Compared to the group without confidence, the group with confidence in getting (*β* = 0.394, OR = 1.483, *p* < 0.001) and affording (*β* = 0.645, OR = 1.906, *p* < 0.001) healthcare when needed had significant positive associations with better SRH. Confidence in both dimensions showed the strongest effect (*β* = 0.715, OR = 2.044, *p* < 0.001). Regarding heterogeneity, affordability-related confidence had particularly strong effects in western regions whereas both dimensions exerted larger effects in rural, compared with urban, areas.

**Conclusion:**

Confidence in the health system—especially in affordability—is strongly associated with better health outcomes in China. Equity-oriented policies that strengthen both accessibility and affordability, with particular attention to disadvantaged regions and vulnerable populations, are essential for improving health equity.

## 1 Introduction

Ensuring healthy lives and promoting well-being for all individuals at all ages is one of the 17 Sustainable Development Goals (SDG). In recent years, considerable advances have been made in improving population health (1). For example, the global life expectancy at birth increased from 66.8 years in 2000 to 73.1 years in 2019, showing a steady increase from the turn of the millennium until the onset of the COVID-19 pandemic (2). At the same time, universal health coverage (UHC)—which ensures that all people can access the full range of quality health services they need, when and where they need them, without financial hardship (3)—has been widely recognized as a critical pathway for improving health outcomes (2). Strengthening health systems and enhancing the sustainability and equity of health services and financing models are thus pivotal for achieving UHC (2).

Despite the crucial role of health systems in improving health outcomes, the uneven distribution of health resources and inequalities in health accessibility and financial risk protection may further widen health disparities. According to the WHO’s analytical framework on priority public health conditions, when health systems deliver services that are less effective or inappropriate for disadvantaged groups, the impact of other social determinants on health outcomes and inequalities may be amplified (4). For instance, in the United States, poorer populations continue to face worse access to care than wealthier groups, partly because many remain uninsured despite the coverage expansions introduced under the Affordable Care Act (ACA) (5). In England, the National Health Service (NHS) has achieved substantial reductions in socioeconomic inequalities in primary care access and quality, yet progress in narrowing inequalities in health outcomes has been modest (6). Globally, efforts to achieve universal health coverage (UHC) have increasingly focused on strengthening health systems as a means to reduce disparities in both access to care and health outcomes (7, 8).

Although the health system provides this institutional context, its effectiveness ultimately depends on how individuals perceive and interact with it. According to the Lancet Global Health Commission on High-Quality Health Systems in the Sustainable Development Goals Era (HQSS), health systems should be judged primarily on their impacts, including improved health and its equitable distribution, public confidence in the health system, economic benefits, and processes of care (9). A high-quality health system offers people a sense of security—or confidence—that they or their family members will receive effective care in case of illness (10). Confidence in the health system is defined as the belief that people can get the healthcare they need if they “become very sick tomorrow” (10). More specifically, it refers to whether individuals in a country are confident that they can obtain and afford good-quality care if they are very sick (11), which represents both the subjective dimension of system performance within the WHO health system framework and an expanded understanding of universal health coverage. This concept has become increasingly important in health policy debates in high-income countries (12) and has more recently attracted attention in low- and middle-income countries (LMIC); however, the evidence remains comparatively limited (10, 13, 14).

As LMICs try to build public support and funding for UHC, research on population confidence in health systems has become particularly important. In addition to understanding the state of confidence in populations, it is essential to examine how confidence relates to the quality of healthcare processes, health outcomes, and the economic benefits of high-quality care (10). Previous research has investigated the extent of confidence in health systems (10, 11) and identified its determinants (14). Building on this foundation, it’s associations with healthcare utilization (15), health insurance enrollment (13), and health status (16), have been explored. These findings suggest that confidence reflects both the supply of health policies and individuals’ experiences, and that it can shape health-seeking behavior and influence outcomes. However, much of this work has focused on service use and insurance coverage, leaving the direct relationship between health system confidence and population health outcomes underexplored.

This gap is particularly salient in low- and middle-income settings, characterized by ongoing health system reforms and persistent structural challenges. China provides a relevant case for such investigation. As a developing country, it has undertaken extensive health reforms in recent years and has achieved notable improvements in population health. Yet, our understanding of how much confidence people have in China’s health system remains limited. Moreover, the association between health system confidence and individuals’ self-reported health warrants further investigation.

Over the past two decades, China has implemented a series of health policy reforms aimed at ensuring equitable and affordable access to quality basic healthcare and providing adequate financial protection (17, 18). Regarding financial risk protection, China achieved universal health insurance coverage (UHIC) by establishing three major social insurance programs: the Urban Employee Basic Medical Insurance (UEBMI, 1998), the New Rural Cooperative Medical Scheme (NRCMS, 2003), and the Urban Resident Basic Medical Insurance (URBMI, 2007). Since 2011, these programs have covered more than 95% of the population. In 2009, the Opinions of the CPC Central Committee and the State Council on Deepening the Health Care System Reform were released to advance the goal of universal access to basic healthcare services and to improve population health. Later, in 2016, the government launched the “Healthy China 2030 Plan,” a national strategy emphasizing equitable, accessible, systematic, and sustainable health services for all (19). During this period, health investment increased considerably, and health expenditure as a share of GDP increased from 5.03% in 2009 to 7.91% in 2023. Simultaneously, the personal health expenditure shared as percentage of total health expenditure declined from 37.46 to 27.33% (20). However, despite rapid policy reforms and rising health investment, China’s pronounced regional disparities mean that resources remain unevenly distributed across areas and population groups (21–24). Consequently, inequalities in health outcomes have become a central concern (25–29).

Building on the concept of health system confidence, this study was conducted with an aim to examine its association with health outcomes in China, with a particular focus on regional disparities. Specifically, we addressed three questions: (1) what are the levels of health system confidence and self-rated health across different regions of China, including urban–rural and east–central–west areas; (2) what is the relationship between confidence in the health system and self-rated health; and (3) do these associations vary across regions?

By addressing these questions, this study contributes to the growing literature on health system confidence in developing countries and provides evidence regarding the impact of this indicator on health outcomes. Furthermore, it identifies priority groups that should be targeted by policies aimed at reducing health inequalities and improving overall population health. Insights from this study may serve as a reference for other LMICs seeking to strengthen equity in their health security systems (30).

## 2 Materials and methods

### 2.1 Data source

The dataset used in this study was sourced from the Chinese General Social Survey (CGSS), the earliest national, comprehensive, and continuous academic survey project in China. The data sources and design reports are available at http://cgss.ruc.edu.cn/English/Home.htm. We used the waves investigated in 2010 and 2021. In these two waves, there is an additional module on EASS (the East Asian Social Survey) health component, which includes questions related to people’s confidence in health system. Using pooled cross-sectional data, an overall sample size of 6,481 was obtained for this study after removing random missing data, with 3,797 in 2010 and 2,684 in 2021.

### 2.2 Measures

The dependent variable in this study was the self-rated health (SRH), which is widely considered and indicator in health-status measurements (26, 27). The SRH indicator was obtained by answering the question, “What do you think of your current health status?” The possible answers were very unhealthy, rather unhealthy, fair, healthy, and very healthy.

Following the People’s Voice Survey on Health System Performance, the core independent variable includes health system confidence, which indicate the confidence to get and afford health services (11), and accordingly, we classified them into accessible confidence and affordable confidence.

In this study, the accessible health system confidence was ascertained by answers to the question, “How do you worry about cannot get health services when needed?” The possible answers were very worried (1), generally worried (2), not worried (3), and totally not worried (4). which are linked to accessibility indicators ranging from low (1) to high (4). Similarly, the affordable health system confidence was determined by answers to the question, “How much do you worry that you cannot afford the health expense when get severe illness?” and the answer contributed to affordability indicators from low (1) to high (4). In the analysis, we combined worry (1) and generally worry (2) into cannot get (cannot afford), and combined not worried (3) and totally not worried (4) into can get (can afford). Furthermore, we combined these two variables into a single variable to obtain a more precise category, including both cannot get and afford (1), can get but cannot afford (2), can afford but cannot get (3), and both can get and afford (4).

The control variables in this study were demographic characteristics, including age, sex, education, and marital status. Furthermore, we controlled for health insurance and area (urban and rural, western, middle, and eastern regions) to minimize the confounding effects of other socioeconomic factors. The definitions, assignments, and descriptive statistics of the variables are presented in Table 1.

**Table 1:** Definition and measurement of variables (*N* = 6,481).

Variables	Number/mean	Percentage/SD
Dependent variable		
Self-related health (SRH)		
Very unhealthy = 1	290	4.47%
Rather unhealthy = 2	906	13.98%
Fair = 3	1,670	25.77%
Healthy = 4	2,205	34.02%
Very healthy = 5	1,410	21.76%
Independent variable		
Accessibility confidence		
Cannot get = 0	4,542	70.08%
Can get = 1	1939	29.92%
Affordability confidence		
Cannot afford = 0	5,138	79.28%
Can afford = 1	1,343	20.72%
Both system confidence		
Both cannot get and afford	4,265	65.81%
Can get but cannot afford	873	13.47%
Can afford but cannot get	277	4.27%
Can both get and afford	1,066	16.45%
Control variable		
Residence		
Rural = 0	2,711	41.83%
Urban = 1	3,770	58.17%
Region		
West area = 0	1,674	25.83%
Middle area = 1	2,689	41.49%
East area = 2	2,118	32.68%
Sex		
Female = 0	3,430	52.92%
Male = 1	3,051	47.08%
Age (years)	55.657	16.684
Eduy (the year of obtaining education, recoded by the type of education)	9.035	4.850
Marital status		
0 = single (includes never married, widowed, or divorced)	1,469	22.67%
1 = married	5,012	77.33%
Health insurance		
0 = no insurance	659	10.17%
1 = insured	5,822	89.83%

SD, standard deviation.

### 2.3 Statistical analysis

General data were summarized using descriptive statistics. An ordered logistic model was used to explore the main factors associated with populations with different SRH. Heterogeneity and interaction-effect analyses were used to explore the different effects among different regions to identify the policy-targeting group. STATA 16.0 was used for the statistical analysis.

## 3 Results

### 3.1 Region-wise health system confidence and SRH status distribution

**Figure 1:**
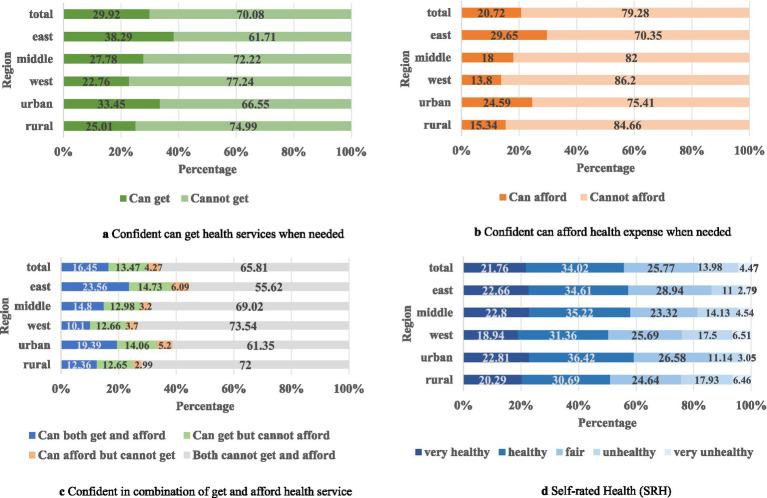
Region-wise distribution of health system confidence and SRH. **(a)** Confident can get health services when needed. **(b)** Confident can afford health expense when needed. **(c)** Confident in combination of get and afford health service. **(d)** Self-rated Health (SRH).

Figure 1 shows health system confidence and SRH status distribution by urban–rural and western–middle–eastern regions. On average, fewer than 20% of the surveyed individuals were confident that they could both obtain and afford healthcare if sick. When comparing these two types of health system confidence, 29.92% of individuals were confident in receiving healthcare, whereas only 20.72% were confident in affording healthcare. Across urban–rural areas, confidence in both getting and affording healthcare when needed was higher in urban areas than in rural areas, together with higher SRH in urban areas than in rural areas. Similarly, across the west–middle–east regions, confidence in getting and affording healthcare when needed was highest at 23.56% in the eastern region, followed by the middle and western regions. Furthermore, this ranking is consistent with other separate health system confidence levels (accessible or affordable). Regarding the SRH status across regions, the highest status was noted in the middle region (58.01%), followed by the eastern region, and was the lowest in the western region. This figure depicts the unequal confidence in the health system among different regions in China; that is, rural and western areas had less accessible and affordable health systems, which may also be linked to health outcome variations.

### 3.2 Results of the ordered logistic model

As the SRH is an ordered variable from 1 to 5, this study employed ordered logistic regression to examine the relationship between health system confidence and health status Based on the definition of health system confidence, we set the heath accessible system confidence dummy [cannot get(compared group) or can get], heath affordable system confidence dummy [cannot afford(compared group) or can afford], both system confidence dummy [both cannot get and afford(compared group), can get but cannot afford, can afford but cannot get, can both get and afford] as three separate independent variables (Model 1 to Model 3). All the models contained control variables and year effects. The key findings are presented in Table 2.

**Table 2:** Results of the ordered logistic model of self-rated health (SRH).

Variables	Model 1	Model 2	Model 3
	SRH	SRH	SRH
Accessible (compared group: cannot get)			
Can get	0.394***		
	(7.73)		
Affordable (compared group: cannot afford)			
Can afford		0.645***	
		(10.99)	
Both system (Compared group: both cannot get and afford)			
Can get but cannot afford			0.105
			(1.54)
Can afford but cannot get			0.474***
			(4.12)
Can both get and afford			0.715***
			(10.92)
Control variable	Control	Control	Control
Year	Control	Control	Control
Cut 1	−5.935***	−5.969***	−5.975***
	(−22.56)	(−22.64)	(−22.66)
Cut 2	−4.251***	−4.279***	−4.284***
	(−16.44)	(−16.52)	(−16.54)
Cut 3	−2.826***	−2.845***	−2.850***
	(−11.01)	(−11.06)	(−11.08)
Cut 4	−1.099***	−1.107***	−1.111***
	(−4.34)	(−4.36)	(−4.37)
*N*	6,481	6,481	6,481
Pseudo *R*^2^	0.0662	0.0694	0.0698

*t*-statistics in parentheses.

***p < 0.001.

Model 1 shows the significant positive association between confidence in getting health care (*β* = 0.394, OR = 1.483, *p* < 0.001) and SRH. Compared with people who do not believe in getting healthcare when needed, confidence in receiving healthcare can increase the odds of reporting higher SRH by 48.3%. Similar to Model 1, Model 2 illustrates the significant positive relationship between confidence in affording healthcare (*β* = 0.645, OR = 1.906, *p* < 0.001) and SRH. Compared to a person who cannot believe in affording healthcare when needed, confidence in affording healthcare can increase the odds of reporting higher SRH by 90.6%; that is, confidence in both accessible and affordable healthcare systems can significantly increase SRH levels. To further explore the comparison effect of different type of confidence, we combined both statements into four categories. When compare with both no confidence in getting and affording healthcare, believing in can get but cannot afford showed no significant effect on SRH; however, low accessible but high affordable health system confidence had a significant positive effect on SRH . (*β* = 0.474, OR = 1.606, *p* < 0.001); besides, confidence in both systems had a higher positive effect on SRH (*β* = 0.715, OR = 2.044, *p* < 0.001).

**Table 3:** Robust test results.

Variables	OProbit Model (total)	Ologit model (year = 2010)	Ologit model (year = 2021)	OProbit Model (total)	Ologit model (year = 2010)	Ologit model (year = 2021)	OProbit Model (total)	Ologit model (year = 2010)	Ologit model (year = 2021)
	SRH	SRH	SRH	SRH	SRH	SRH	SRH	SRH	SRH
Accessible (compared group: cannot get)									
Can get	0.236***	0.415***	0.344***						
	(7.92)	(5.84)	(4.58)						
Affordable (compared group: cannot afford)									
Can afford				0.379***	0.645***	0.607***			
				(11.06)	(7.83)	(7.07)			
Both (compared to: both cannot)									
Can get but cannot afford							0.0724^+^	0.156	0.0338
							(1.82)	(1.63)	(0.34)
Can afford but cannot get							0.284***	0.509***	0.362*
							(4.22)	(3.31)	(2.06)
Can both get and afford							0.421***	0.720***	0.671***
							(11.02)	(7.75)	(7.05)
Control variable	Control	Control	Control	Control	Control	Control	Control	Control	Control
Year	Control	/	/	Control	/	/	Control	/	/
*N*	6,481	3,797	2,684	6,481	3,797	2,684	6,481	3,797	2,684
Pseudo *R*^2^	0.0670	0.0719	0.0605	0.0701	0.0745	0.0643	0.0705	0.0748	0.0646

*t*-statistics in parentheses.

^+^*p* < 0.10, *p < 0.05, ***p < 0.001.

### 3.3 Robust test

We conducted a series of robustness checks to confirm the reliability of our findings. The first robustness check replaces the initial-order logistic regression model with an order probit model. This methodological switch allowed us to assess the sensitivity of our results to alternative estimation techniques, ensuring that our conclusions were not influenced by specific assumptions inherent in the logistic framework. Furthermore, we performed a second robustness check by analyzing samples from different years. As the health policy changed significantly from 2010 to 2021, this resulted in different levels of health system confidence. By examining regressions across these distinct timeframes, we determined whether our results remain consistent despite variations in the policy development period. The results of both robustness checks indicate a high degree of stability and affirmed the validity of our initial findings, and thereby reinforced the reliability of our conclusions regarding the impact of health system confidence on SRH (Table 3).

### 3.4 Heterogeneity analysis

To examine regional heterogeneity in the association between health system confidence and SRH, we employed subsample regressions across urban and rural settings as well as the eastern, western, and central regions. Moreover, we constructed interaction terms between urban and rural classification and health system confidence, as well as between regional classifications and health system confidence. Therefore, we explored how variations in this relationship might exist in different regional groups in order to identify priority areas as targets of health policy. The key results are presented in Tables 4–6.

**Table 4:** Heterogeneity in the association between health system confidence and SRH (accessible confidence).

Variables	Rural	Urban	West	Middle	East	Interaction urban–rural	Interaction region
	SRH	SRH	SRH	SRH	SRH	SRH	SRH
Accessible (compared group: cannot get)							
Can get	0.513***	0.357***	0.458***	0.371***	0.401***	0.540***	0.487***
	(6.16)	(5.54)	(4.28)	(4.52)	(4.80)	(6.48)	(4.55)
Urban						0.167**	
						(2.82)	
Can get*urban						−0.201+	
						(−1.94)	
Middle							0.302***
							(4.56)
East							0.360***
							(4.94)
Can get*middle							−0.110
							(−0.83)
Can get*east							−0.123
							(−0.92)
Control variable	Control	Control	Control	Control	Control	Control	Control
Year	Control	Control	Control	Control	Control	Control	Control
*N*	2,711	3,770	1,674	2,689	2,118	6,481	6,481
Pseudo *R*^2^	0.0624	0.0633	0.0652	0.0662	0.0662	0.0648	0.0661

The subsample regressions and interaction analysis of urban–rural variations does not control for west-middle-east region, and the analysis of middle-east region variations does not control for urban–rural. West, rural are set as compared groups.

*t*-statistics in parentheses.

^+^*p* < 0.10, **p < 0.01, ***p < 0.001.

**Table 5:** Heterogeneity in the association between health system confidence and SRH (affordable confidence).

Variables	Rural	Urban	West	Middle	East	Interaction urban–rural	Interaction region
	SRH	SRH	SRH	SRH	SRH	SRH	SRH
Affordable (compared group: cannot afford)							
Can afford	0.849***	0.589***	0.826***	0.632***	0.633***	0.899***	0.879***
	(8.40)	(8.19)	(6.15)	(6.53)	(7.03)	(8.89)	(6.62)
Urban						0.156**	
						(2.81)	
Can afford*urban						−0.346**	
						(−2.85)	
Middle							0.303***
							(4.85)
East							0.358***
							(5.20)
Can afford*middle							−0.228
							(−1.41)
Can afford*east							−0.333*
							(−2.11)
Control variable	Control	Control	Control	Control	Control	Control	Control
Year	Control	Control	Control	Control	Control	Control	Control
*N*	2,711	3,770	1,674	2,689	2,118	6,481	6,481
Pseudo *R*^2^	0.0665	0.0668	0.0691	0.0691	0.0708	0.0685	0.0696

The subsample regressions and interaction analysis of urban–rural variations does not control for west-middle-east region, and the analysis of middle-east region variations does not control for urban–rural. West, rural are set as compared groups.

*t*-statistics in parentheses.

*p < 0.05, **p < 0.01, ***p < 0.001.

**Table 6:** Heterogeneity in the association between health system confidence and SRH (both system confidences).

Variables	Rural	Urban	West	Middle	East	Interaction urban–rural	Interaction region
	SRH	SRH	SRH	SRH	SRH	SRH	SRH
Both (compared group: both cannot)							
Can get but cannot afford	0.250*	0.0102	0.207	0.0716	0.0370	0.263*	0.233+
	(2.34)	(0.11)	(1.53)	(0.67)	(0.31)	(2.44)	(1.71)
Can afford but cannot get	0.908***	0.310*	0.821**	0.388+	0.404*	0.961***	0.911***
	(4.29)	(2.25)	(3.19)	(1.91)	(2.39)	(4.50)	(3.56)
Can both get and afford	0.890***	0.666***	0.869***	0.705***	0.705***	0.939***	0.916***
	(7.91)	(8.28)	(5.72)	(6.57)	(6.98)	(8.34)	(6.06)
Urban						0.194**	
						(3.21)	
Can get but cannot afford *urban						−0.253+	
						(−1.83)	
Can afford but cannot get*urban						−0.683**	
						(−2.71)	
Can both get and afford*urban						−0.306*	
						(−2.26)	
Middle							0.326***
							(4.81)
East							0.382***
							(5.06)
Can get but cannot afford*middle							−0.166
							(−0.96)
Can get but cannot afford*east							−0.182
							(−1.02)
Can afford but cannot get*middle							−0.484
							(−1.49)
Can afford but cannot get*east							−0.600*
							(−1.97)
Can both get and afford*middle							−0.198
							(−1.08)
Can both get and afford*east							−0.292
							(−1.63)
Control variable	Control	Control	Control	Control	Control	Control	Control
Year	Control	Control	Control	Control	Control	Control	Control
*N*	2,711	3,770	1,674	2,689	2,118	6,481	6,481
Pseudo *R*^2^	0.0672	0.0674	0.0696	0.0694	0.0712	0.0691	0.0701

The subsample regressions and interaction analysis of urban–rural variations does not control for west-middle-east region, and the analysis of middle-east region variations does not control for urban–rural. West, rural are set as compared groups.

*t*-statistics in parentheses.

^+^*p* < 0.10, *p < 0.05, **p < 0.01, ***p < 0.001.

On average, the findings reveal that, in both western and rural regions, the positive effects of confidence in the health system on health outcomes were significantly amplified. More specifically, Table 4 shows the relationship between confidence in receiving healthcare and SRH in different areas. For the urban–rural difference, the rural area exhibits the strongest effect of health accessible confidence on SRH (*β* = 0.513, OR = 1.670, *p* < 0.001), the interaction model also confirmed this result. When introduced urban–rural*can get (reference: rural and cannot get) confidence interaction, the significant negative coefficient for urban*can get indicates that weaker confidence on accessible health system effectiveness in urban areas than in rural areas (*β* = −0.201, OR = 0.818, *p* < 0.1); thus, this effect is larger in rural areas. Although the subgroup analysis results were significant in different regions, with the largest coefficient in the western area, followed by the eastern and middle areas, this moderate effect was not significant.

Table 5 shows the relationship between confidence in affording healthcare and SRH status in different areas. Similar to confidence in an accessible health system, the effect among urban–rural differences were stronger in rural areas than in urban areas, and the interaction model showed a smaller effect in urban areas. However, this result differed in west–middle–east regions, compared to the western region, the result of the interaction between the eastern region and can afford health system confidence showed a smaller effect (*β* = −0.333, OR = 0.717, *p* < 0.1).

Table 6 shows the relationship between confidence in both obtaining and affording healthcare and SRH in different areas. Referring to the both cannot get and afford groups, the high accessibility confidence with low affordability confidence shows a significant positive effect on SRH in rural areas but without significant effects in other places. However, regarding the high affordability confidence with low accessibility confidence, both health system confidences showed significant positive effects in all areas, with rural areas achieving larger effects than urban areas and the western region obtaining a larger effect than the middle and eastern regions. When employing the interaction analyses, referring to both no confidence and every health system confidence type showed a smaller effect in urban areas than in rural areas; however, the region (west–middle–east) moderation was only significant among those with high affordable health system confidence.

## 4 Discussion

This study examined the association between confidence in health system and self-rated health (SRH) in China, with a particular focus on regional differences. The three main findings that emerged deepen our understanding of health system confidence and provide insights for health policy development in developing countries.

First, the overall confidence in the health system remains relatively low and exhibits substantial regional variation. Fewer than 20% of respondents across regions reported being somewhat or very confident in their ability to obtain and afford needed care if they became ill. This aligns with previous studies on health system confidence in China (10, 14), which have highlighted persistent public concerns about the reliability and fairness of health policy and resource distribution. When comparing different dimensions of confidence, we found a pattern which is consistent with cross-national evidence that shows affordability tends to generate weaker confidence than accessibility (11). The low reported confidence in obtaining and affording care suggests that the existing health insurance does not cover the desired services or that covered services do not meet people’s quality expectations (11). Moreover, regional analysis revealed that residents in western and rural areas consistently reported lower levels of confidence. These results indicate that despite the achievement of universal insurance coverage and improvements in financial protection, people in China continue to face concerns regarding financial barriers and service quality. Thus, they still face a high incidence of catastrophic expense together with the unequal health policy development and health resource distribution in subnational areas (21–24, 31).

Secondly, higher confidence in the health system is positively associated with better SRH, with the effect of affordability-related policies being particularly pronounced. This result is similar to a previous study which showed that financial protection and perceived quality play significant roles in determining public perception, whereas accessibility has limited influence on people’s perception of people’s perception of China’s health system (31). Thus, equitable access to basic primary healthcare services is a key step in establishing a tiered healthcare system for all (24), which is also a fundamental human right and a pillar of a country’s sustainable development (32). Moreover, financial protection policies, such as health insurance, reduce the risk of illness-induced poverty. Both accessibility and affordability can encourage healthcare utilization and ultimately improve health outcomes. More importantly, the combination of accessibility and affordability must be noticed. Although health services are available, they may not translate into improved health outcomes unless they are also affordable to patients. In addition, whereas the expansion of benefit package in the domain of financing is indeed a decisive move toward universal health coverage, the essential financial protection of the poor cannot be achieved without strong and coordinated supply-side reforms that target cost containment (33).

Finally, the positive association between confidence in the health system and SRH is particularly pronounced in disadvantaged regions, such as the western and rural areas. This pattern aligns with prior studies which showed that health insurance integration confers more benefits on vulnerable groups, including older adults, rural residents, and rural-to-urban migrants (34). One possible explanation is that these disadvantaged regions face inherently weaker health resources, which makes additional resource investment and institutional support more impactful. Although the China health insurance system covers more than 95% of the residents, there are certain groups—such as migrants, informal sector workers, and some rural residents—that remain excluded or are intermittently enrolled owing to increasing premiums. Besides, the benefits are unevenly distributed among different areas or between the labor sector and non-workers. For instance, according to the National Healthcare Security Administration, in 2024, the hospitalization reimbursement rate reached 84.8% under employee health insurance but only 68.6% under resident insurance (35). From the perspective of diminishing marginal returns, policy interventions and resource allocations frequently yield greater health benefits when directed toward resource-constrained regions and vulnerable populations. These results highlight the need for equity-oriented policies that explicitly prioritize disadvantaged groups.

The results of this study reveal future directions for further improving China’s health system and the other developing countries. First, given the overall low confidence in the health system—particularly regarding affordability—it is imperative to strengthen the health system as a whole, and achieving truly universal health insurance coverage should be prioritized. With equitable access being one of the main priorities of the health insurance, more efforts will be required to reach the poorest groups (13). In practice, this means that policies promoting insurance reimbursement in under-resourced regions should be carefully designed to ensure that vulnerable groups are not left behind.

In addition, since confidence in both accessibility and affordability are associated with better health outcomes, combining accessibility improvements with affordability safeguards could yield synergistic benefits, which can increase the health status of the entire population and reduce health inequality among different regions. For example, China’s policy of immediate reimbursement for cross-regional treatments helps the floating population achieve access to care while reducing their financial burden, and thereby addresses both accessibility and affordability dimensions to narrow health disparities (36). Additionally, continued reforms to hospital incentives and integrated delivery systems are critical for controlling health expenditures and enhancing healthcare quality (31).

Finally, the heterogeneous association between health system confidence and SRH across regions highlights the importance of spatially targeted health investments. This indicates that health policies should be implemented in different areas according to local resources and health needs. More importantly, these efforts should especially target to vulnerable groups. In western and rural areas, strengthening primary healthcare services and ensuring universal social protection coverage should be prioritized, complemented by financial transfer subsidies to bridge regional gaps. In contrast, eastern and urban regions could focus on reducing government responsibility while encouraging diversified, market-based healthcare investments.

Despite the insights gained from this study, it is important to acknowledge its limitations. First, owing to data constraints, we relied on mixed cross-sectional rather than panel data, and this limits causal inference (16) and prevents an analysis of how health policy reforms affect the same groups over time. In addition, our measures of confidence in accessibility and affordability, as well as SRH, were based on brief survey questions and reflect subjective perceptions; thus, a more precise measurement may consist of various questions related to health system confidence (15). Finally, the mechanisms through which confidence influences SRH remain insufficiently understood. Although we identified associations and heterogeneity, further research is needed to clarify the pathways linking confidence to health outcomes, including the role of contextual, behavioral, and institutional factors. Longitudinal studies of specific groups or regions, as well as refined classifications of health system types, would strengthen the understanding of these dynamics and provide a more reliable basis for policymaking under regional disparities.

## 5 Conclusion

This study examined the association between confidence in the health system and SRH from a regional comparative perspective. We found that overall confidence in the health system was relatively low and varied significantly across regions, with the lowest levels observed in western and rural areas. Higher levels of confidence were positively associated with better SRH, particularly when related to affordability, and this association was strongest in disadvantaged regions. These findings suggest that policies enhancing both affordability and accessibility are critical for improving population health and reducing regional inequalities. Targeting vulnerable groups with equity-oriented reforms should be prioritized to strengthen confidence in the health system and to advance health equity.

## Data Availability

Publicly available datasets were analyzed in this study. The datasets analyzed for this study can be found in the website of The Chinese General Social Survey (CGSS): http://cgss.ruc.edu.cn/English/Home.htm.

## References

[1] United Nations. Goal 3: ensure healthy lives and promote well-being for all at all ages. Available online at: https://www.un.org/sustainabledevelopment/health/ (accessed September 01, 2025).

[2] World Health Organization. World health statistics 2024: Monitoring health for the SDGs, sustainable development goals. Geneva: World Health Organization (2024).

[3] World Health Organization Universal health coverage (UHC) (2025). Available online at: https://www.who.int/news-room/fact-sheets/detail/universal-health-coverage-(uhc) (accessed September 01, 2025).

[4] World Health Organization. Equity, social determinants and public health programmes. Geneva: World Health Organization (2012).

[5] Dickman SL Himmelstein DU Woolhandler S. Inequality and the health-care system in the USA. Lancet. (2017) 389:1431–41. doi: 10.1016/S0140-6736(17)30398-7, PMID: 28402825

[6] Asaria M Ali S Doran T Ferguson B Fleetcroft R Goddard M. How a universal health system reduces inequalities: lessons from England. J Epidemiol Community Health. (2016) 70:637–43. doi: 10.1136/jech-2015-206742, PMID: 26787198 PMC4941190

[7] Wagstaff A Neelsen S. A comprehensive assessment of universal health coverage in 111 countries: a retrospective observational study. Lancet Glob Health. (2020) 8:e39–49. doi: 10.1016/S2214-109X(19)30463-2, PMID: 31837954

[8] Atun R de Andrade LO Almeida G Cotlear D Dmytraczenko T Frenz P. Health-system reform and universal health coverage in Latin America. Lancet. (2015) 385:1230–47. doi: 10.1016/S0140-6736(14)61646-9, PMID: 25458725

[9] Kruk ME Gage AD Arsenault C Jordan K Leslie HH Roder-DeWan S. High-quality health systems in the sustainable development goals era: time for a revolution. Lancet Glob Health. (2018) 6:e1196–252. doi: 10.1016/S2214-109X(18)30386-3, PMID: 30196093 PMC7734391

[10] Roder-DeWan S Gage A Hirschhorn LR Twum-Danso NAY Liljestrand J Asante-Shongwe K. Level of confidence in and endorsement of the health system among internet users in 12 low-income and middle-income countries. BMJ Glob Health. (2020) 5:e002205. doi: 10.1136/bmjgh-2019-002205, PMID: 32859647 PMC7454186

[11] Kruk ME Kapoor NR Lewis TP Arsenault C Boutsikari EC Breda J. Population confidence in the health system in 15 countries: results from the first round of the people’s voice survey. Lancet Glob Health. (2024) 12:e100–11. doi: 10.1016/S2214-109X(23)00499-0, PMID: 38096882 PMC10716625

[12] Gilson L. Trust in health care: theoretical perspectives and research needs. J Health Organ Manag. (2006) 20:359–75. doi: 10.1108/14777260610701768, PMID: 17087400

[13] Osei Afriyie D Masiye F Tediosi F Fink G. Confidence in the health system and health insurance enrollment among the informal sector population in Lusaka, Zambia. Soc Sci Med. (2023) 321:115750. doi: 10.1016/j.socscimed.2023.115750, PMID: 36801748

[14] Zhao D Zhao H Cleary PD. Understanding the determinants of public trust in the health care system in China: an analysis of a cross-sectional survey. J Health Serv Res Policy. (2019) 24:37–43. doi: 10.1177/1355819618799113, PMID: 30176742

[15] Musa D Schulz R Harris R Silverman M Thomas SB. Trust in the health care system and the use of preventive health services by older black and white adults. Am J Public Health. (2009) 99:1293–9. doi: 10.2105/AJPH.2007.123927, PMID: 18923129 PMC2696665

[16] Mohseni M Lindstrom M. Social capital, trust in the health-care system and self-rated health: the role of access to health care in a population-based study. Soc Sci Med. (2007) 64:1373–83. doi: 10.1016/j.socscimed.2006.11.023, PMID: 17202025

[17] Yip W Fu H Jian W Liu J Pan J Xu D. Universal health coverage in China part 1: Progress and gaps. Lancet Public Health. (2023) 8:E1025–34. doi: 10.1016/S2468-2667(23)00254-2, PMID: 38000882

[18] Yip W Fu H Jian W Liu J Pan J Xu D. Universal health coverage in China part 2: addressing challenges and recommendations. Lancet Public Health. (2023) 8:E1035–E1042. doi: 10.1016/S2468-2667(23)00255-4, PMID: 38000883

[19] Ning C Pei H Huang Y Li S Shao Y. Does the healthy China 2030 policy improve people’s health? Empirical evidence based on the difference-in-differences approach. Risk Manag Healthc Policy. (2024) 17:65–77. doi: 10.2147/RMHP.S439581, PMID: 38204928 PMC10778192

[20] National Bureau of Statistics of China. China Statistical Yearbook 2024. (2024). Available online at: https://www.stats.gov.cn/sj/ndsj/2024/indexeh.htm (accessed September 01, 2025).

[21] Yuan L Cao J Wang D Yu D Liu G Qian Z. Regional disparities and influencing factors of high quality medical resources distribution in China. Int J Equity Health. (2023) 22:8. doi: 10.1186/s12939-023-01825-6, PMID: 36627636 PMC9832614

[22] Huang X. Four worlds of welfare: understanding subnational variation in Chinese social health insurance. China Q. (2015) 222:449–74. doi: 10.1017/S0305741015000399

[23] Huang M Rozelle S Cao Y Wang J Zhang Z Duan Z. Primary care quality and provider disparities in China: a standardized-patient-based study. Lancet Reg Health West Pac. (2024) 50:101161. doi: 10.1016/j.lanwpc.2024.101161, PMID: 39253593 PMC11381900

[24] Jia P Wang Y Yang M Wang L Yang X Shi X. Inequalities of spatial primary healthcare accessibility in China. Soc Sci Med. (2022) 314:115458. doi: 10.1016/j.socscimed.2022.115458, PMID: 36279792

[25] Cai J Coyte PC Zhao H. Decomposing the causes of socioeconomic-related health inequality among urban and rural populations in China: a new decomposition approach. Int J Equity Health. (2017) 16:128. doi: 10.1186/s12939-017-0624-9, PMID: 28720105 PMC5516311

[26] Shang XT Wei ZH. Socio-economic inequalities in health among older adults in China. Public Health. (2023) 214:146–52. doi: 10.1016/j.puhe.2022.11.013, PMID: 36549024

[27] Tang Y Fu R Noguchi H. Impact of medical insurance integration on reducing urban–rural health disparity: evidence from China. Soc Sci Med. (2024) 357:117163. doi: 10.1016/j.socscimed.2024.117163, PMID: 39121565

[28] Yuan L Yu B Gao L Du M Lv Y Liu X. Decomposition analysis of health inequalities between the urban and rural oldest-old populations in China: evidence from a national survey. SSM Popul Health. (2023) 21:101325. doi: 10.1016/j.ssmph.2022.101325, PMID: 36618546 PMC9816804

[29] Yao Q Zhang X Wu Y Liu C. Decomposing income-related inequality in health-related quality of life in mainland China: a national cross-sectional study. BMJ Glob Health. (2023) 8:e013350. doi: 10.1136/bmjgh-2023-013350, PMID: 38035731 PMC10689391

[30] He W. Social medical insurance integration and health care disparities in China: evidence from an administrative claim dataset. Econ Anal Policy. (2023) 79:20–39. doi: 10.1016/j.eap.2023.05.023

[31] Zhu Y Li Y Wu M Fu H. How do Chinese people perceive their healthcare system? Trends and determinants of public satisfaction and perceived fairness, 2006-2019. BMC Health Serv Res. (2022) 22:22. doi: 10.1186/s12913-021-07413-0, PMID: 34983522 PMC8725557

[32] Chowdhury JJ Ravi RP. Healthcare accessibility in developing countries: a global healthcare challenge. J Clin Biomed Res. (2022) 4:1–5. doi: 10.47363/JCBR/2022(4)152

[33] Liu K Zhang Q He AJ. The impacts of multiple healthcare reforms on catastrophic health spending for poor households in China. Soc Sci Med. (2021) 285:114271. doi: 10.1016/j.socscimed.2021.114271, PMID: 34352505

[34] Huo J Hu M Li S. The impact of urban–rural medical insurance integration on medical impoverishment: evidence from China. Int J Equity Health. (2023) 22:245. doi: 10.1186/s12939-023-02063-6, PMID: 37996948 PMC10668423

[35] National Healthcare Security Administration. (2024). National Statistical Bulletin on healthcare security advancement. Available online at: https://www.nhsa.gov.cn/art/2025/7/14/art_7_17248.html (accessed September 01, 2025).

[36] Tian J Chen Z Wang Y Zhu Y. Does the trans-provincial immediate reimbursement reduce health gap between urban and rural floating population? Evidence from China. BMC Public Health. (2025) 25:1826. doi: 10.1186/s12889-025-23027-1, PMID: 40382571 PMC12084940

